# The role of long non-coding RNA in abdominal aortic aneurysm

**DOI:** 10.3389/fgene.2023.1153899

**Published:** 2023-03-15

**Authors:** Yi Xu, Shuofei Yang, Guanhua Xue

**Affiliations:** Department of Vascular Surgery, School of Medicine, Renji Hospital, Shanghai Jiao Tong University, Shanghai, China

**Keywords:** abdominal aortic aneurysm (AAA), epigenetic modifications, non-codingRNA, longnon-codingRNA, smooth musclecells

## Abstract

The abdominal aortic aneurysm (AAA) is characterized by segmental expansion of the abdominal aorta and a high mortality rate. The characteristics of AAA suggest that apoptosis of smooth muscle cells, the production of reactive oxygen species, and inflammation are potential pathways for the formation and development of AAA. Long non-coding RNA (lncRNA) is becoming a new and essential regulator of gene expression. Researchers and physicians are focusing on these lncRNAs to use them as clinical biomarkers and new treatment targets for AAAs. LncRNA studies are beginning to emerge, suggesting that they may play a significant but yet unidentified role in vascular physiology and disease. This review examines the role of lncRNA and their target genes in AAA to increase our understanding of the disease’s onset and progression, which is crucial for developing potential AAA therapies.

## 1 Introduction

AAAs are characterized by a weakening and dilation of the abdominal aorta’s wall and typically affect the infrarenal portion of the abdominal aorta (Sakalihasan et al., 2005). Although other definitions are available, such as aortic diameter at 50% of the normal diameter, AAA is most defined as having an inferior renal abdominal aorta with a maximum diameter of more than 30 mm on ultrasound or CT imaging (Moll et al., 2011). The main epidemiological risk factors for AAA are being male, older than 65, and having a smoking history. Smoking is the most significant and modifiable risk factor (Golledge, 2019). Atherosclerosis, high blood pressure, race, and a family history of AAAs are additional risk factors. Quitting smoking reduces the risk of AAA and inhibits aneurysm growth. The most dangerous complication of a AAA is aneurysm rupture. Inadequate treatment can result in a death rate of up to 65%–85% (Moll et al., 2011).

lncRNAs are the focus of research and clinical efforts to investigate their potential as clinical biomarkers and new treatment targets for AAAs. However, the significance of lncRNAs in vascular physiology and disease is still unknown because research on them is still in its infancy. Therefore, in addition to discussing the mechanisms underlying AAA and the potential diagnostic and therapeutic use of lncRNAs, this review examines the role of lncRNAs and their target genes in AAA.

Due to the lack of therapeutic interventions to stop aneurysm dilatation or prevent aneurysm growth, a significant emphasis must be placed on expanding our understanding of the underlying mechanisms of AAA. From a therapeutic standpoint, a better understanding of epigenetic processes can aid in developing pharmacological therapies to maintain the aorta wall and halt disease progression. It can also help prevent life-threatening consequences such as ruptured AAAs.

## 2 Current theories of AAA pathogenesis

The pathophysiology of AAAs is extraordinarily complex, involving vascular smooth muscle cell death, inflammatory cell infiltration, oxidative stress, and extracellular matrix breakdown. In addition, a combination of biochemical, gene, immunological, and other factors promotes the progression of AAA.

### 2.1 Death and phenotypic switch of vascular smooth muscle cells

Vascular smooth muscle cells (VSMC), a crucial blood vessel type, can produce extracellular matrix (ECM) components such as elastin and collagen. The decreased density of VSMC in the aortic medium is an apparent pathological characteristic of aneurysms (López-Candales et al., 1997), and apoptosis is the likely culprit (Henderson et al., 1999). A decrease in VSMCs is observed in AAA, which may lead to a decrease in the ECM and a weakening of the aortic wall. In AAA, VSMCs have a dead form, primarily apoptosis (Quintana and Taylor, 2019). Several factors can promote the apoptosis of VSMCs. When macrophages infiltrate the aortic aneurysm wall, they express many inflammatory factors that can promote VSMC-apoptosis, such as interleukin 6 (IL-6), tumor necrosis factor-α (TNF-*α*), and monocyte chemotaxis protein-1 (MCP-1) (Hsieh et al., 2001). Moreover, LDL can promote apoptosis. Transforming growth factor-*β* (TGF-β) can inhibit apoptosis and protect AA. In addition, VSMCs experience ER stress due to cytokine and mechanical stretch stimulation, resulting in programmed cell death (Jia et al., 2017). Therefore, targeting the apoptosis of VSMCs is one of the therapeutic approaches for AA. The apoptosis-inhibiting transcription factor EB (TFEB) is downregulated in AA patient tissues. Hydroxypropyl-β-cyclodextrin (h-β-cd) can activate TFEB, inhibit AA, and be a potential clinical therapeutic agent (Lu et al., 2020). In the process of AAA, many matrix metalloproteases (MMPs) accelerate the breakdown of the extracellular matrix (ECM), triggering the normal growth limitation of VSMC and exacerbating VSMC death. ECM generated by VSMC itself plays a role in supporting cell development. The apoptotic cycle finally takes shape. Receptor-interacting protein kinase three signals in AAA tissues contributed to the progression of the disease by inducing necrosis and apoptosis of VSMC, which in turn triggered vascular inflammation (Wang et al., 2015a).

During the formation of the embryonic vasculature, smooth muscle progenitors are recruited to the vascular network composed of endothelial cells and then influenced by cytokines such as platelet-derived growth factor-BB (PDGF-BB) and TGF-*β*, which differentiate into mature VSMCs. The ascending and descending aortas have distinct origins (Wang et al., 2015b). VSMCs in the ascending aorta develop from the second heart field and the cardiac neural crest, whereas VSMCs in the descending aorta develops from the mesodermal lineage. This distinction distinguishes TAA from AAA. Four VSMC is highly plastic and can switch between the two phenotypes. The systolic type has a significant VSMC phenotype, whereas the synthetic type has dedifferentiated properties. The actile type is essential for the aortic wall and maintenance of aortic strength. In instances of inflammation and injury, VSMCs switch to a synthetic phenotype with strong proliferative and migratory capacity and increased secretion of fibrosis-associated and inflammation-related proteins, such as increased expression of osteopontin and decreased expression of contractile proteins (Owens, 1995). A phenotypic shift is what this process is known as. Numerous cardiovascular diseases, including atherosclerosis and hypertension, have been linked to VSMC phenotypic switching in addition to AA. TGF-*β*, which affects cell differentiation, proliferation, and apoptosis, is one of the most extensively studied growth factors (Gadue et al., 2006). The prevalent belief is that TGF-*β* is a protective factor for AAA. However, it has also been suggested that excessive inhibition of VSMC proliferation may weaken the aortic wall structure and result in AA expansion. In addition, TGF-*β* was significantly upregulated in AA individuals. Thus, the role of TGF-*β* remains contentious (Doyle et al., 2015).

### 2.2 Infiltration of inflammatory cells

Chronic abdominal vascular wall inflammation causes AAA. Chronic inflammation is a major pathogenic characteristic of AAA. Numerous inflammatory cells, including T cells, macrophages, dendritic cells, neutrophils, B cells, mast cells, and even VSMC, infiltrate the aorta wall, indicating that these cells are essential to the occurrence and progression of AAA (Yuan et al., 2021). The progression of AAA is one of these, and macrophages and lymphocytes are two of the most important contributors.

M1 and M2 are the two primary macrophage subtypes. After activation, M1-type macrophages are drawn to damaged arteries by increased production of proteolytic enzymes and several inflammatory cytokines, which exacerbate local inflammation and help in aortic dilatation and vascular remodeling (Raffort et al., 2017). M1-type macrophages can produce MMP-9 and MMP-3 to aid in the breakdown of the ECM, and their production of TNF and IL-1 induces VVSMCs to secrete MMPs (Furusho et al., 2018). On the other hand, M2-type macrophages can regulate angiogenesis, cell recruitment, and collagen deposition, all of which are advantageous for ECM remodeling and tissue healing. As AAA progressed, M1-type macrophages gave way to M2-type macrophages in the aorta wall.

Most of the lymphocytes in AAA tissues, which include T and B lymphocytes, are CD4^+^ T cells (Zhou et al., 2013). TNF- and IFN-secreting, macrophage-stimulating, and collagen-inhibiting Th1 cells are all present. The expression of Fas ligand (FasL) by Th2 cells may facilitate the apoptosis of VSMC. The IL-17 released by Th17 cells exacerbates the oxidative stress in the abdominal aortic wall and promotes neutrophil recruitment. The production of IFN- by CD8^+^ T cells can stimulate apoptosis and the recruitment of macrophages that produce MMP *in vivo*.

Neutrophils also play a significant role in AAA development. Some advantages to neutrophil inflammation are brought on by the formation of neutrophil extracellular traps (NETs) and the release of neutrophils to combat infections (Wang et al., 2015a). However, NETs that develop too frequently can harm the body and contribute to the pathophysiology of cardiovascular diseases (Janiuk et al., 2021). The development of a AAA can be accelerated by NETs, which can not only directly damage the abdominal aortic wall but also stimulate the production of IL-6 and IL-1 in macrophages, increase Th17 cell differentiation, and attract other inflammatory cells (Papayannopoulos, 2018). According to MeherAK et al., IL-1 primarily induces the formation of NETs during AAA formation (Meher et al., 2018).

Inflammasomes also play a role in the emergence and progression of AAA. Studies have connected the NLRP3 and AIM2 inflammasomes to the pathophysiology of AAA. According to Wu et al., the normal NLRP3 inflammasome complex promotes the degradation of SMC contraction protein in a caspase-1-dependent manner, resulting in AAA. According to studies, individuals with AAA had higher levels of the AIM2 inflammasome in their peripheral immune cells, suggesting that this enzyme is involved in the immunological or inflammatory response to AAA (Wu et al., 2017a). In addition, AIM2’s interaction with cytoplasmic DNA activates the inflammasome, releasing IL-1 (Man et al., 2016).

### 2.3 Oxidative stress

Local oxidative stress is one of the established fundamental causes of abdominal aortic inflammation, and oxidative stress is the primary mechanism causing damage to the abdominal aortic wall. Oxidative stress results from a competitive imbalance between free radicals and antioxidants, which relates primarily to produce reactive oxygen species (ROS) by activating pro-MMP2 and pro-MMP9, which degrade collagen fibers in artery walls (Sakalihasan et al., 2018). Similarly, ROS can increase the production of ROS and MCP-1, which facilitate monocyte penetration into blood vessel walls. The urokinase-type plasminogen activator, which promotes VSMC growth, can activate the ROS gene in VSMC (McCormick et al., 2007). In addition, Nicotinamide Adenine Dinucleotide Phosphoate Oxidase must be active for endothelial Nitric Oxide Synthase to synthesize AAA (Siu et al., 2017). Due to its dose-dependent protective effect against VSMC oxidative stress and angiotensin II-induced apoptosis in AAA mouse models, apelin may have a therapeutic effect on AAA (Wang et al., 2019).

Interactions between oxidative stress and inflammation can exacerbate the damage to arterial tissue. In chronically inflamed tissues, elevated ROS levels are frequently observed. It has been demonstrated that inflammatory mediators can activate NADPH oxidase to produce peroxide ions and that both NADPH oxidase and iNOS are involved in the inflammatory response. ROS products encourage inflammatory cell infiltration and increase the secretion of pro-inflammatory cytokines. In addition to these effects, ROS directly activates MMPs (Anand et al., 2018) and inhibits the MMP inhibitor plasminogen activator inhibitor type 1 (PAI-1) (Bobadilla et al., 2010) to induce VSMC apoptosis.

### 2.4 Degradation of extracellular matrix

The degradation of extracellular matrix ECM, composed of elastin, collagen, and other components, is essential for maintaining the vascular homeostasis of the abdominal aorta. The breakdown of ECM, essentially the breakdown of elastic and collagen fibers in the middle abdominal aorta, leads to the development of AAA and the remodeling of the arterial wall. MMP and how ECM degrades are closely related. MMP-2, MMP-9, and other MMPs are essential in AAA. MMP-2 and the early expansion of AAA have a close relationship (Nagase et al., 2006), whereas MMP-9 and the ongoing expansion and rupture of AAA have a close relationship (Li et al., 2020). Following endovascular repair, MMP-9 can be used as a circulating marker to predict the development of aneurysms and endoleakage (Maleux et al., 2017). The interaction between MMP-9 and MMP-2 facilitates the expansion and rupture of AAA (Fontaine et al., 2002). Additionally, the synthetic VSMC has a high MMP release capacity. Oxidative stress and inflammation can stimulate MMP release, accelerating ECM degradation.

### 2.5 Luminal thrombus

Most clinically significant AAAs contain a complex structure of fibromin, inflammatory cells, platelets, and red blood cells in the aneurysm sac, known as the luminal thrombus (ILT), and antithrombotic therapy has become a potential agent for treating the progression of AAA (Adolph et al., 1997).

It is becoming increasingly clear that platelets, in addition to their roles in hemostasis and thrombosis, are involved in the pathological process of vascular disease. AAA is a form of atherosclerotic thrombotic disease characterized by the formation of non-occlusive ILT, whose thromboinflammatory state contributes to outward remodeling and ultimately disrupts aortic wall integrity. Platelet activation is involved in AAA pathogenesis through membrane receptors and the secretory mediator (Harter et al., 1982). The formation of a thrombus in the ILT and the accumulation of inflammatory cells and cytokines can compromise the structural integrity and stability of the vessel wall, increasing the risk of dissection and rupture (Satta et al., 1996). Disordered blood flow in the aneurysmal sac also promotes platelet activation and aggregation.

The ILT is often structured in three layers in AAA patients, referred to as the luminal (proximal to the blood), medial (intermediate region), and abluminal (proximal to the aneurysm wall) regions (Gersh et al., 2009). Platelets, neutrophils, and erythrocytes are abundant in the luminal ILT layer that comes into contact with blood. The ILT is rarely embolized but does not subside once it occurs (Behr-Rasmussen et al., 2014). The eccentric distribution of ILT is associated with the continuous expansion of AAA, with thicker ILT volumes growing. Inflammatory cells and cytokines are said to accumulate in the ILT and play an essential role in the progression of AAA (Touat et al., 2006). Due to the lack of vascularization in the middle aorta, oxygen obtained by luminal diffusion, or “luminal pole hypoxia,” can exacerbate inflammation, fibrinolysis, and cell death in the adjacent aortic wall, resulting in progressive thinning and subsequent expansion of the canal wall (Piechota-Polanczyk et al., 2015). It suggests that thrombosis is bioactive and contributes to the evolution of AAA patho genes. One study found a correlation between ILT thickness and MMP-9 expression (Khan et al., 2012). Roxana et al. reported that local C3 retention, depletion, and proteolysis in the ILT could induce chemotaxis and activation of polymorphonuclear leukocytes and decrease the later stages’ systemic complement concentration and activity of AAA (Martinez-Pinna et al., 2013). Craig N. Morrell et al. found that transcriptomic analysis of platelets from AAA patients revealed upregulation of signal transduction pathways shared by olfactory receptors, which is thought to mediate AAA progression (Morrell et al., 2022).

ILT has both beneficial and detrimental effects on the progression or rupture of AAA. However, it is now believed that its harmful ones outweigh its protective effects (Vande Geest et al., 2008). ILT has been demonstrated to reduce AAA’s peak wall stress, protect the arterial wall, and prevent rupture. On the other hand, ILT may act as an inflammatory focal point for proteolysis and enzymatic degeneration of the aortic wall, increasing the risk of rupture (Haller et al., 2018). Furthermore, we and others have demonstrated that its damaging biochemical properties obscure the biomechanical protective properties of ILT in small AAAs.

### 2.6 Endothelial dysfunction

A thick layer of ILT associated with endothelial disruption is a characteristic of almost all AAAs (Franck et al., 2013). Although the association between ILT and the progression of AAA has historically attracted interest and research, the effects of endothelial cell (EC) destruction and altered endothelial phenotype in the context of AAA have yet to be thoroughly investigated (Widmer and Lerman, 2014).

ECs play a significant role in regulating vascular homeostasis and blood flow. Vascular tone, angiogenesis, wound healing, smooth muscle cell proliferation, fibrosis, and inflammation are all regulated by ECs. In addition, ECs are essential for maintaining non-thrombotic blood-tissue interfaces with limited permeability. Endothelial dysfunction has been shown to trigger various vascular diseases, including AAA. ECs promote AAA expansion by increasing oxidative stress, nitric oxide (NO) bioavailability, adhesion molecule expression, and recruitment by inflammatory cells (Sun et al., 2018). The pathological changes of endothelial cells or endothelial dysfunction may precede the pathological changes of the media and adventitia during AAA formation.

Lack of eNOS increased atherosclerosis and AAA in apoE^−/−^ mice but did not affect arterial blood pressure, body weight, serum cholesterol concentration, or lipoprotein cholesterol distribution (Kuhlencordt et al., 2001). No bioavailability regulates endothelial cell function, and its production is regulated by eNOS expression and coupling status (Lu et al., 2016).

Unidirectional laminar flow shear stress (LSS) and oscillatory shear stress are two types of shear stresses produced by the influence of different flow modes on the vessel wall (OSS). High LSS inhibits the development of AAA, which has been reported to occur in areas of reflux, low wall shear stress, or blood flow disturbances (Wu et al., 2022). Hemodynamic regulation of oxidative stress and reactive oxygen species (ROS) in endothelium LSS inhibits superoxide production in endothelial cells, whereas OSS increases superoxide production in endothelial cells. In addition, NOX mediates oxidative stress in response to various shear stresses. LSS induces NO production by activating NOX 2, whereas OSS activates NOX 1 by decoupling eNOS. OSS demonstrated increased MMP activity and decreased TIMP-3 expression compared to LSS (Blann et al., 1998). Endothelial cells at the site of the AAA lesion showed increased expression of cathepsins (Calvayrac et al., 2011). The expression and activity of cathepsins in endothelial cells were significantly lower under laminar flow than in turbulent flow (Vasa et al., 2001). By controlling inflammatory cell infiltration and releasing inflammatory factors, hemodynamics indirectly mediates the expression of cathepsins in endothelial cells.

Endothelial cells are also involved in oxidative stress in the aortic wall. Many genes linked to inflammatory and oxidative responses in the aortic wall appear mediated by the transcription factor nuclear factor κB (NF-κB) (Wolf et al., 1994). It was discovered that when the NF-κB pathway of endothelial cells is blocked, the infiltration of inflammatory cells in the arterial media and adventitia, inflammatory factor expression, and the oxidative stress response decrease; this inhibits aneurysm formation (Satta et al., 1996). Endothelial cell oxidative stress occurs before VSMCs are altered, and inflammatory cells are infiltrated. Endothelial cells are stimulated by secreted materials that increase ROS levels in smooth muscle. Increased levels of ROS in endothelial cells alter the function of vascular smooth muscle cells (VSMCs) and promote oxidative stress, in which cyclophilin A (CypA) may act as an intermediate molecule (Cameron et al., 2018). CypA induces smooth muscle cell migration and proliferation, increases the expression of endothelial adhesion molecules, and mediates chemotaxis in inflammatory cells.

### 2.7 PVAT

PVAT plays a vital role in vascular function and disease (Kugo et al., 2016). PVAT is a continuous anatomic structure in direct contact with the adventitia of the vessel. It can regulate arterial homeostasis by adjusting dilation and contractile functions, producing soluble factors with autocrine or paracrine effects, and modulating inflammation. PVAT dysfunction is frequently associated with atherosclerotic and non-atherosclerotic vascular diseases and is thought to participate in AAA pathogenesis (Queiroz and Sena, 2020).

Genome-wide expression profiling by Luca Piacentini et al. reveals the autoimmune response signature of perivascular adipose tissue (Piacentini et al., 2019). PVAT activates inflammatory signals and stimulates immune cell recruitment and activation. Immune cells produce soluble factors that start matrix degradation, sustained by the constant activation of innate and adaptive immune responses. Tissue damage, cell death, and autoantigen exposure result from persistent proteolytic imbalance. It dramatically amplifies autoimmune responses by different pathways involving AAA initiation or progression. Mast cells may play a crucial role in the early stages. Initial aneurysm development may be characterized by Class II-MHC antigen presentation by antigen-presenting cells (APC), differentiation of type 2 T helper cells (Th2), and humoral immune response. Typically, as AAA progresses, the adaptive immune response shifts to antigen presentation *via* cytotoxic responses mediated by class-I MHC and T cells. Other factors like aging and gene factors may also contribute to immune response dysfunction and AAA progression. Flow cytometry shows that T cells are most prevalent in the PVAT in AAA. CD4^+^ and CD8^+^ T cell populations are highly activated in both compartments, with the CD4^+^ T cell population exhibiting the highest activation state within the AAA wall (Sagan et al., 2019).

In the vascular wall of AAAs, cytokines such as MCP-1 and c-reactive protein (CRP) are highly secreted in PVAT, promoting VSMC proliferation, elastic arterial stiffness, and accelerating aneurysm formation (Furusho et al., 2018). PVAT-mediated angiopoietin-like protein 2 (Angptl2) increases MMP-2 and MMP-9 expression (Horimatsu et al., 2017). Researchers have demonstrated that inhibiting MMP secretion in PVAT may reduce AAA size, indicating that the progression of AAAs can be controlled by modulating PVAT-derived MMP. Transcriptomic analysis revealed that PDGF-D is strongly expressed in the PVAT of obese mice; inhibition of PDGF-D function significantly reduced the incidence of AAA (Zhang et al., 2018). Experiments indicate that adipocyte-specific PDGF-D transgenic mice are more likely to develop AAA, ECM fibrosis, and inflammation.

PVAT is dysfunctional in pathological states and plays a significant role in AAA formation. PVAT-derived factors are involved in all phases of pathological AAA formation, including inflammatory cell infiltration, oxidative stress onset, matrix metalloproteinase activation, and VSMC phenotypic switching. Therefore, PVAT may be a valuable new target for developing therapeutic agents for AAA. Interestingly, Huang et al. discovered that PVAT in the thoracic aorta inhibited vascular smooth muscle cell apoptosis and abdominal aortic aneurysm formation in mice (Huang et al., 2023).

## 3 The role of epigenetic modifications in abdominal aortic aneurysm pathogenesis

**FIGURE 1:**
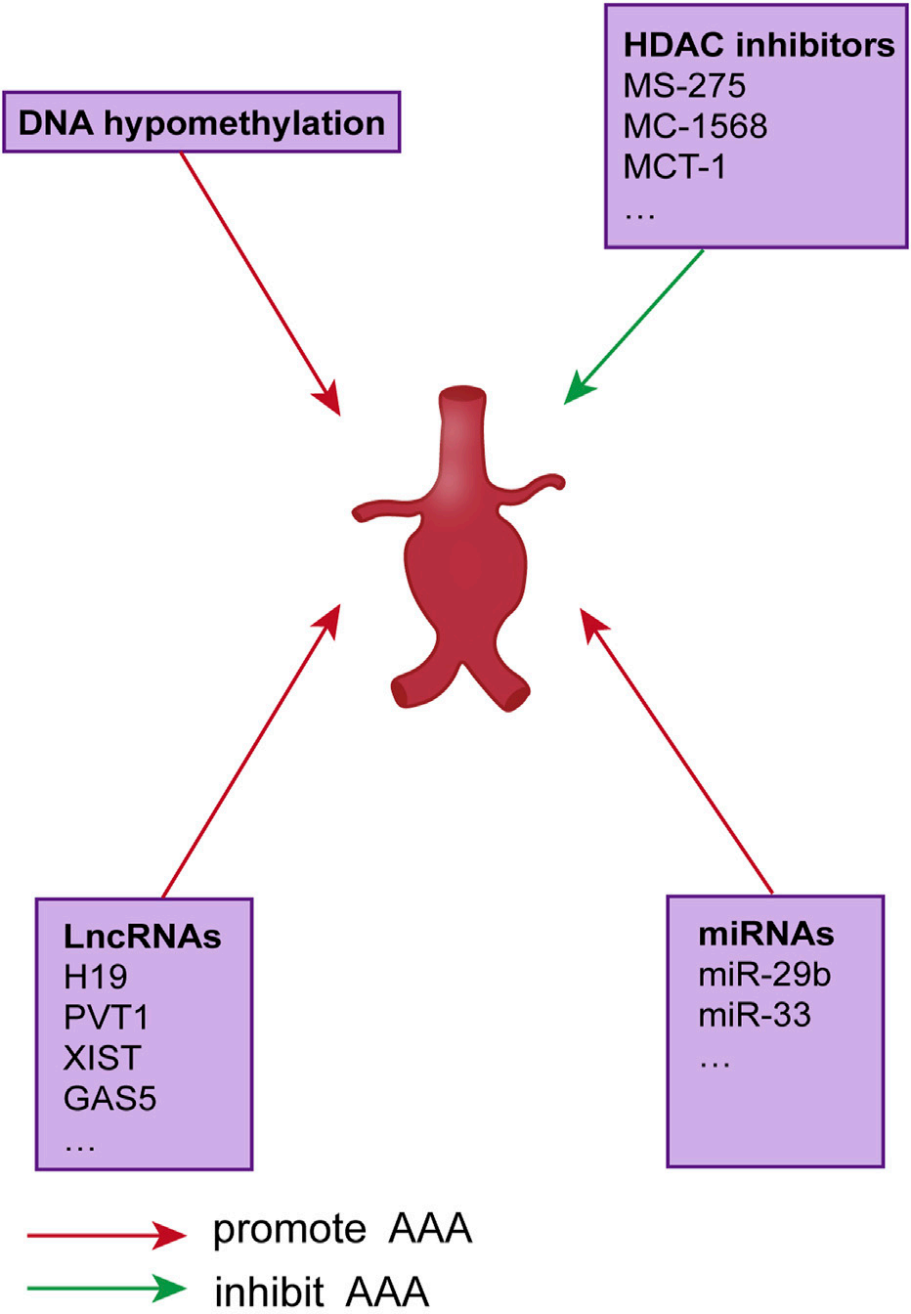
Epigenetic regulation of AAAS.

Epigenetic modifications are developmental or environmental changes that do not alter the genetic code but regulate how DNA-encoded information is expressed in a tissue- and environment-specific manner. As with the genetic cause of disease, epigenetic mechanisms lead to dysregulation of aneurysm gene expression without significant sequence variation. DNA methylation, post-translational histone modifications, and non-coding RNA comprise the three main categories of epigenetic regulation patterns. All of these regulate gene transcription. Some of these epigenetic changes have an established role in the pathogenesis of AAA genes; however, in most cases, the relationship between epigenetic regulation and aneurysm development is unclear (Figure 1).

### 2.1 DNA methylation

DNA methylation is an inherited epigenetic modification that occurs in CpG islands within the gene promoter, leading to transcriptional silencing, and is performed on cytosines by DNA methyltransferases (DNMTs). According to studies, the development of AAA is driven by T cell dysfunction, specifically decreased inhibition of CD4^+^CD25+T regulatory cells (Yin et al., 2010) This change is partly caused by epigenetic alterations such as DNA methylation, with significantly less methylation in T cells in AAA patients than in healthy controls (Toghill et al., 2015). Similarly, the expression of DNMT 1, DNMT3A, and methyl-CpG-binding domain 2 (MBD 2), both involved in DNA methylation and transcriptional repression, was decreased in T cells isolated from AAAs patients and mediating DNA methylation (Xia et al., 2019).

Several additional studies suggest that hypermethylation of specific gene promoters is also associated with the occurrence of AAA and DNA hypomethylation. Recent studies have characterized surgical samples and animal models to demonstrate the role of epigenetic modifications of monocytes and macrophages in the pathogenesis of aortic aneurysm formation (Toghill et al., 2018). Global methylation levels in peripheral blood mononuclear cells were significantly altered in the CpG island and positively correlated with increased aortic diameter in AAA patients compared to controls. Single-cell RNA sequencing of human AAA tissue revealed increased expression of the histone demethylase JMJD 3 in aortic monocytes and macrophages, resulting in an upregulation of the inflammatory immune response (Davis et al., 2021). Mechanistically, interferon-β regulates the expression of JMJD 3 *via* JAK/STAT, and JMJD 3 induces nfkb-mediated transcription of inflammatory genes in infiltrating aortic macrophages. Pharmacological or gene therapy inhibition of macrophage-specific JMJD 3 can stop the development and rupture of AAA.

### 2.2 Histone modification

Depending on specific histone marks, depending on histone marks, histone modifications in key promoter and enhancer gene regions are associated with gene activation or repression. Histone acetylases, deacetylases, and methyltransferases all perform these modifications. The binding of essential transcription factors that can activate or repress gene transcription is promoted or prevented by the combination of these histone marks, which alters the structure of chromatin. Additionally, unique histone features can differentiate between diseased and healthy tissues.

In one study, for instance, the expression of several histone deacetylases (HDAC) was elevated in AAA samples compared to healthy samples (Han et al., 2016) The HDAC expression of ApoE and AAA mouse models was also elevated. The HDAC expression of Ang II infused ApoE and AAA mouse models was also higher than that in controls. In the same study, HDACs’ expression, particularly classes I and IIa, increased in human AAA samples compared to control samples and colocalized with macrophages in the aortic wall. Using a mouse model, treatment with class I (MS-275) or class IIa (MC-1568) HDAC inhibitors decreased the incidence of AAA, macrophage inflammation, and pro-inflammatory mediators, partly due to decreased MMP-2 and MMP-9 activity following HDAC inhibition (Galán et al., 2016). In addition, MCT-1 decreased the expression of MMP-2 in vSMCs and MMP-2 and MMP-9 in aortic tissues (Vinh et al., 2008). Previous research suggests that the MMP-2 and MMP-9 genes contain histone acetylation patterns that control AAA formation and that focusing on these sites may prevent AAA from spreading.

### 2.3 Non-coding RNA

Since only 1–2 percent of the human genome codes for proteins and many non-coding regulatory elements are translated into non-coding RNAs (ncRNAs), ncRNAs may play complex and vital regulatory roles in higher animals. Non-coding RNAs include lncRNAs and short non-coding RNAs (sncRNAs), which include siRNAs, miRNAs, and piRNAs. Less conserved than ncRNAs, lncRNAs regulate gene expression in poorly understood ways. Long non-coding RNA (lncRNA) refers to transcribed RNA molecules longer than 200 nucleotides. We will talk about lncRNA in the content but concentrate on miRNAs here. miRNAs are 17–23 bases short single-stranded RNA molecules that exert multiple cellular functions by directly regulating gene expression. In general, miRNAs negatively regulate gene expression by degrading mRNA or inhibiting translation. In either case, the mature miRNA is paired with a highly conserved “seed” sequence within the 30 UTR of the target gene. Notably, a single miRNA can regulate multiple genes; consequently, most miRNAs are involved in complex gene expression regulatory networks. miRNA-29 regulates fibrotic responses and ECM *in vitro* and *in vivo*, thereby promoting the formation of AAA, according to multiple studies (Maegdefessel et al., 2012). Several collagen types in the aorta, including COL1A1, COL2A1, COL3A1, and COL5A1, are effective targets of miRNA-29b (Maegdefessel et al., 2014a). The miRNA-29 also targets the ECM genes ELN and FBN1 and the members of the MMP family (Boon et al., 2011). The expression of fibrotic genes like COL1A1, COL3A1, and EL3 was found to be decreased when miRNA-29b was overexpressed, according to Maegdefessel et al. In contrast, miRNA-29b inhibition with anti-miR-29b increased fibrotic gene expression and decreased AAA incidence.

In addition, miR-155 levels were significantly higher in AAA samples than in controls (Biros et al., 2014). It has been demonstrated that miRNA-155 is essential in promoting CTLA 4. CTLA 4 promotes chronic inflammation by enhancing T-cell development by downregulating cytotoxic T-lymphocyte-associated proteins.

It has also been demonstrated that miRNA regulates macrophage function. For example, direct targeting of Chi311 by miRNA-24 reduces inflammation by inhibiting the recruitment and survival of macrophages and the production of the cytokines IL-8 and CCL 2 by SMCs and macrophages (Maegdefessel et al., 2014b). Another important miRNA is miR-33, a lack of which reduces “M1” gene expression, protease activity, and macrophage infiltration into the aortic wall (Nakao et al., 2017). In addition, bone marrow transplantation of miR-33-deficient cells decreased AAA formation, indicating that bone marrow-specific miR-33 may contribute to the pathogenesis of AAA genes.

## 4 Functions of lncRNAs

According to chromosomal locations or DNA strand features, lncRNA can be further classified as suitable, antisense, intragenic, intergenic, enhancer, or cyclic, among other categories. In addition, according to studies, lncRNA can act as a signal, decoy, guide, and skeleton molecule to control gene expression.

### 4.1 lncRNA functions as a signaling molecule

The majority of lncRNAs are transcribed by RNA polymerase II. Because each lncRNA’s transcription has a unique spatiotemporal expression pattern, it can function as a molecular signal to integrate better developmental signals, cell contents, and responses to environmental cues (Devaux et al., 2015). Some of these lncRNAs have regulatory functions, while others are transcriptional products. lncRNA can play a role in regulating the initiation, progression, and termination of transcription. In these circumstances, the only factor affecting the chromatin state of regulatory elements is the expression of connected lncRNAs. LncRNAs, which serve as signaling molecules, can also identify developmental and temporal phases and regulate gene expression. *Xist* is a non-coding RNA essential for the inactivation of the X chromosome. XistRNA is expressed on the inactive X chromosome during the development of female animals, and it is then superimposed on the X chromosome for transcription (Brockdorff et al., 2020). As a result, the X chromosome is inactivated, and gene expression on it is suppressed due to the extensive methylation of histones at this time. The antisense transcription factor Tsix inhibits *Xist* expression, whereas the accumulation of the non-coding RNA Jpx on the inactive X chromosome increases *Xist* expression.

### 4.2 lncRNA functions as a decoy molecule

Promoter or enhancer transcription significantly impacts the transcriptional regulation of lncRNA. LncRNAs that serve as booby traps are transcribed and bind to specific protein targets but have no other activities. These RNAs serve as “molecular filters” that promote the binding of RNA-binding proteins, which can be transcription factors, chromatin repairers, or other types of regulators (Fatica and Bozzoni, 2014). A lncRNA known as Gas5 (crowtharrest-specific 5) was said to have been discovered. Gas5 may cause cells to develop glucocorticoid resistance. In the promoter region of glucocorticoid response genes, Gas5 prevents glucocorticoid receptors from forming RNA motifs through one of the stem-ring structures, which are analogous to DNA motifs that are analogs of hormone-responsive elements (Filippova et al., 2021). Consequently, Gas5 serves as a dummy molecule that competitively binds to the glucocorticoid receptor’s DNA binding site, effectively blocking the receptor’s ability to interact with chromosomes.

### 4.3 lncRNA functions as a guide molecule

lncRNAs can guide RNA-binding proteins to help the ribosomal protein complex localize to specific locations. Recent studies have demonstrated that lncRNAs can mediate changes in gene expression *via* cis (neighboring genes) or trans (distant genes) (Loewen et al., 2014). According to the regulatory role that these RNAs play at the transcriptional level, changes in chromatin structure may have structural effects that are not only local but also distant. For instance, lncRNAs like Air or eRNAs can significantly impact the environment thanks to local sequence elements like promoters and enhancers that are transcriptionally controlled. Long-distance gene regulation for lncRNAs, such as HOTAIR and linc-p21, requires additional interacting components, which must be precisely positioned at the site of action (Angrand et al., 2015). Lastly, lncRNAs can function as complementary targets for microRNAs in a cis-acting manner or co-transcribe to influence chromatin modification further. When used as RNA-DNA heterologous double-stranded nucleic acid molecules, such as RNA, DNA, the DNA triple complex, or an RNA recognition feature on a particular chromatin surface, LncRNAs attach to target DNA to influence chromatin modifications in a trans-regulatory manner.

Some lncRNAs regulate gene expression differently than these cis-regulated lncRNAs; they do so by trans-acting, which means they extend their transcriptional impact on genes across chromosomes. It has been observed that lncRNA HOTAIR expression and cancer metastasis are related. Both metastatic and primary breast cancers exhibit elevated HOTAIR expression (Qu et al., 2019). In addition, cancer cells with high PRC2 expression became less aggressive due to HOTAIR deficiency. These findings suggest that non-coding RNA-mediated polycomb complexes play an essential role in the development of breast tumors. lncRNAs such as HOTAIR can further regulate the epigenetic state of cells by focusing on the location of chromatin repair complexes and the activity of their enzymes.

### 4.4 lncRNA functions as a skeleton molecule

To further regulate gene expression, lncRNA can serve as a hub for the recruitment of molecular players in relevant biological processes. This precise control is essential for the unique and dynamic chemical interactions and signals transduction processes involved in various biological activities. This kind of lncRNA is comparatively complicated since it interacts with several effector molecules and has various functional areas (Statello et al., 2021). Moreover, by introducing effector proteins with transcriptional activation or inhibition, lncRNAs can bind to multiple effector molecules simultaneously.

The interaction between lncRNA and the chromatin repair complex significantly facilitates the inhibition of INK4a gene transcription. According to studies, the polycomb complex-mediated methylation of H3K27 influences the expression of the tumor suppressor subsite INK4b/ARF/INKa antisense non-coding RNA in both healthy and malignant cells (Aguilo et al., 2011). ANRIL can interact directly with the PRC1 and PRC2 complex components and shares the exact location as INK4b. If ANRIL disrupted any interaction with PRC1 or PRC2, it would affect the transcriptional repression of INK4b target genes. Therefore, ANRIL is a typical scaffold protein that recruits multiple chromatin repair complexes to mute target genes and dynamically regulates gene transcriptional activity.

## 5 AAA and lncRNAs

Few studies have examined the function of lncRNAs in the onset and progression of vascular disease. Currently, H19 is the only lncRNA associated with AAA (Li et al., 2018). Numerous studies have discovered that lncRNAs play a crucial role in the processes required to grow and spread AAA. However, only a few studies listed below have demonstrated *in vivo* evidence that the lncRNA mediates the presumed molecular mechanism of action. Further research is necessary to determine if lncRNAs other than H19 are equally as effective as miRNAs in determining the fate of aortic aneurysms. Here, we briefly review current research on aortic aneurysms using lncRNAs(Table 1).

**TABLE 1:** Summary of lncRNAs regulating pathophysiology of AAAs.

**LncRNA**	**Targets**	**Functions**	**Reference**
H19	miR-675, HIF1α	Generates miRNA; promotes VSMC proliferation; induces VSMC apoptosis	Wang et al. (2015a)
PVT1	MMP-2/9	promotes apoptosis and phenotype switching	Wu et al. (2017a), Wu et al. (2022)
GAS5	miR-21	inhibits SMC proliferation and apoptosis	Zhang et al. (2018)
XIST	miR-29b,miR-762	promotes VSMC apoptosis	Zhao et al. (2018), Zou et al. (2021)

### 5.1 H19

Li et al. identified mouse lncRNA H19 by RNA sequencing AngII and PPE-induced AAAs in mice. Inhibition of H19 inhibits aneurysm formation in AngII- and PPE-AAA mouse models, low-ldlr Yucatan mini pigs, samples from AAAs patients, and low-ldlr Yucatan mini pigs (Li et al., 2018). H19 is markedly elevated in advanced AAAs.

VSMC proliferation and migration were negatively correlated with rising H19 expression levels, whereas apoptosis was positively correlated. Enhanced H19 binds to the HIF1 promoter region in the nucleus of VSMCs and recruits the transcription factor Sp1, increasing HIF1 expression. H19 acts as a scaffold to inhibit pro-survival signaling by interacting with the HIF-1 protein and keeping it in the cytoplasm. Increased cytoplasmic HIF-1 directly interacts with MDM2 and blocks the MDM2-mediated decrease of p53, causing BAX and BCL-2 levels to rise and fall, respectively. It increases AAA growth and progression by inducing VSMC apoptosis.

Mechanistically, HIF1-*α* (hypoxia-inducible factor 1-*α*) was identified as the primary target of H19. The combination of HIF1-α and H19 increased VSMC death. In addition, the interaction between H19 and HIF1-*α* increased the amount and activity of p53 in the cytoplasm (Sun et al., 2019). H19 attracts the SP1 transcription factor to the promoter region in the nucleus to initiate HIF1-transcription. lncRNA H19 also contributes to AAA development by increasing IL-6 and MCP-1 levels and macrophage infiltration in the aorta. The H19/let-7aceRNA pathway contributes to this IL-6-promoting effect.

### 5.2 GAS5

Cell division, apoptosis, differentiation, and growth arrest are just a few of the biological processes to which lncRNA GAS5 is functionally linked. Recent research has identified GAS5 as a crucial regulator that safeguards the proliferative/migratory phenotype of vascular VSMCs. Several studies have identified the protein scaffold for regulating p21 and phosphatase and tensin homolog (Tao et al., 2017), which are linked to SMC proliferation and apoptosis during aneurysm development, as a downstream target of GAS5 in several investigations. One of the most prevalent and severely dysregulated miRNAs in several cardiovascular illnesses is miR-21. It has been shown that this miRNA plays a crucial role in the development of AAA. GAS5 was significantly elevated in VSMCs from human and mouse AAA models. By preventing cell growth, inducing SMC apoptosis, and inhibiting GAS5 signaling, GAS5 overexpression accelerated the development of AAA in a mouse model. The release of PTEN from miR-21-mediated regulation by GAS5 acts as a sponge for miR-21, preventing Akt phosphorylation and activation. Additionally, Y-box binding protein 1 (YBX 1) and GAS5 collaborate to create a positive feedback loop that encourages the production of p21 downstream (He et al., 2019). On the other hand, vascular smooth muscle cells showed less apoptosis when GAS5’s long non-coding RNA was knocked down. GAS5 may also act in AAA *via* EZH-2-dependent RIG-I augmentation. Therefore, a viable treatment approach for AAA may involve the GAS-5/EZH2/RIG-I axis (Le et al., 2021).

### 5.3 PVT1

PVT1 is a recently discovered lncRNA with oncogenic molecular functions in gastrointestinal, cervical, pancreatic (Zhao et al., 2018), and non-small cell lung cancer (Wu et al., 2017b). PVT1 was shown to be elevated more than thrice in AAA tissues compared to normal tissues using microarray analysis, demonstrating a direct connection between PVT1 and AAA pathogenesis. Intriguingly, the murine AAA model also revealed that inihibited functions might be a prime therapeutic target for AAA prevention, according to Huang et al., who discovered that ncRNA PVT1 is involved in AAA progression by sequestering miR-3127-5p and increasing the expression of its target NCKAP1L (Huang et al., 2021). Moreover, in a mouse model of AAA, the deletion of lncRNA PVT1 prevented the death of VSMCs and the breakdown of the extracellular matrix (Miyagawa et al., 2017).

### 5.4 XIST

lncRNA XIST is a chromosome Xq13.2 transcript that influences the activation of genes associated with the X chromosome. As a ceRNA, XIST contributes to development of multiple diseases, including non-small cell lung cancer, liver cancer, and triple-negative breast cancer (Lan et al., 2021). High-throughput sequencing revealed that XIST expression was elevated in human thoracic aortic dissection, providing additional evidence that XIST is associated with thoracic aortic dissection. Zhang et al. (Haller et al., 2018) found that patients with Stanford A aortic dissection had significantly higher XIST expression in the aortic wall tissue (Zhang et al., 2020). By regulating the miR-29b-3p/Eln signaling pathway, XIST causes apoptosis in arterial smooth muscle cells in thoracic aortic aneurysms (Liang et al., 2021). According to Zou et al., AAA patients’ serum levels of XIST are significantly expressed (Zou et al., 2021). We discovered that XIST was consistently elevated in Ang II-treated VSMCs and AAA mice produced by Ang II. In Ang II AAA mice, XIST knockdown stimulated cell division while preventing apoptosis. An essential pathophysiology of AAA is VSMC apoptosis (Zhang et al., 2019). Consequently, XIST may influence AAA formation by regulating VSMC apoptosis.

Zhang et al. discovered that the XSMT/miR-762/MAP2K4 axis contains the chemical MAP2K4, which is involved in the apoptosis of VSMCs. The inhibition of XIST increased miR-762 expression, decreased MAP2K4, MMP-2, and MMP-9 protein expression, and significantly slowed the development of mouse AAA. MMP-2 and MMP-9 degrade elastin and collagen, and their absence in rats inhibits the development of aneurysms (Miyagawa et al., 2017). In addition, miR-1264, regulated by the WNT/-catenin signaling pathway, is one mechanism by which XIST inhibited apoptosis and promoted the proliferation of HA-VSMCs. As a result, XIST could be helpful in the fight against AAA.

## 6 Perspectives

Recent research has demonstrated conclusively that non-coding RNAs, such as lncRNA, play a role in the occurrence and progression of AAA. Although numerous lncRNAs have suggested potential roles in specific pathologic pathways of AAA development, H19 is the only lncRNA that unequivocally establishes its function in AAA formation. Therefore, the need for lncRNA mechanistic investigations and their significance in AAA are extensive. A better understanding of the processes of AAA provided by this information will aid in the development of therapeutic and diagnostic methods. To understand the molecular and pathophysiological mechanisms by which these non-coding RNAs control the development and progression of AAA, it is crucial to identify and confirm the aberrantly expressed LNCRNAs in sick human tissues and associated animal models.

Once the expression of these lncRNAs has been confirmed, the molecular mechanisms of these lncRNAs should be investigated *in vitro* and *in vivo* in various cell types. The control of RNA is the most crucial and challenging aspect of determining the molecular mechanism of these molecules. The complexity and diversity of lncRNA’s three-dimensional regulatory mechanisms prevent the development of simple target gene prediction methods. The primary focus of investigation should be on the genes that are integrated into, around, or surrounding the target lncRNA genome. In addition to being therapeutically targeted, lncRNAs can be used as biomarkers to track the progression of AAA in patients. In conclusion, many non-coding RNAs have been identified as crucial regulators of AAA, and this list is growing. However, additional research is required to identify additional players, explain their mode of action, and determine their diagnostic and therapeutic potential.
